# Diphtheria

**Published:** 1893-03

**Authors:** 


### DIPHTHERIA.

Concerning the management of a case of diphtheria, so far as that may fall within the domain of the parents, the following few rules, while not incorporating all, are still the most important for preventing the spread of this dreadful disease, and my earnest advice to every mother is to study them carefully, and preserve them for future reference.

First, strips of linen or cotton fabric, about eight inches wide, folded several times, and long enough to reach from ear to ear, should be rung out of ice water (if in winter) and if in summer put directly upon ice, and then applied externally directly to the throat, and as fast as one cloth gets warm another should be ready to take its place. If the child complains of being cold, its feet and hands should be bathed in as hot water as it can stand. When the child is very young, it may be readily ascertained if it be cold or not by feeling its hands and head. Under no circumstances should hot applications be made to the throat. If the child is old enough it may be given broken ice to suck constantly, even if the water is spit out. The cold applications inhibit the growth of the microbes. The patient's hands should be washed frequently—and here let me say so should those of attendants—and the vessel used for the purpose should not be used by any one else. The patient's clothing needs protection in front. This may be done by pinning back of the neck a large piece of linen or cotton fabric, which will cover the whole front of the child and reach as far as the knees. A material should be used which can easily be boiled or burned when soiled. The little patient, if old enough, will want to spit, and for a spittoon a small wooden box with an inch of sawdust on the bottom, is capital. Fresh sawdust should be supplied at least once a day—three times a day would be better—and that which has been used should be emptied upon a good, hot fire, and thus burned at the time the change is made. If there are any flies about, the box should be kept covered, and, as a matter of course, only uncovered when the patient desires to spit; otherwise, the flies alighting upon this spittle would carry the germs of the disease with them, and then alighting upon the family's food and drink, necessarily infect them, and thus indirectly infect the whole family. This is by no means chimerical, but a well established fact.

All clothing and bed clothing that come in contact with a diphtheria patient contain the germs of the disease. For this reason all such clothing should be disinfected, and aired or washed before it is used again.

What is said of diphtheria is also true of scarlet fever and measles. The contagion of these diseases is in proportion to the severity of the case from which it comes. The less care given to ventilation and general cleanliness, the more active does the contagion become; but the contagion from a very mild case may cause very severe and fatal cases.

Both diphtheria and scarlet fever may be caused by milk poisoned with the germs of these diseases. There are on record at least fourteen epidemics of diphtheria caused by milk.

In only one of these epidemics did any of the attendants about the dairies have diphtheria before the disease broke out among the customers. In one instance it broke out among the dairy hands and the customers at the same time. In five of these epidemics the dairies were in very unsanitary conditions; untrapped or open drains allowed noxious gases to reach the milk and milk pans. In this way a small amount of the contagion got into the milk and developed so rapidly as to infect a great many people.

